# Multibatch TMT Reveals False Positives, Batch Effects and Missing Values*

**DOI:** 10.1074/mcp.RA119.001472

**Published:** 2019-07-22

**Authors:** Alejandro Brenes, Jens Hukelmann, Dalila Bensaddek, Angus I. Lamond

**Affiliations:** Centre for Gene Regulation and Expression, School of Life Sciences, University of Dundee, Dow St, Dundee, DD1 5EH, United Kingdom

**Keywords:** Tandem mass spectrometry, mass spectrometry, computational biology, data evaluation, bioinformatics, data analysis, false positives, ipsc, isobaric tags

## Abstract

Multiplexing strategies for large-scale proteomic analyses have become increasingly prevalent, tandem mass tags (TMT) in particular. Here we used a large iPSC proteomic experiment with twenty-four 10-plex TMT batches to evaluate the effect of integrating multiple TMT batches within a single analysis. We identified a significant inflation rate of protein missing values as multiple batches are integrated and show that this pattern is aggravated at the peptide level. We also show that without normalization strategies to address the batch effects, the high precision of quantitation within a single multiplexed TMT batch is not reproduced when data from multiple TMT batches are integrated.

Further, the incidence of false positives was studied by using Y chromosome peptides as an internal control. The iPSC lines quantified in this data set were derived from both male and female donors, hence the peptides mapped to the Y chromosome should be absent from female lines. Nonetheless, these Y chromosome-specific peptides were consistently detected in the female channels of all TMT batches. We then used the same Y chromosome specific peptides to quantify the level of ion coisolation as well as the effect of primary and secondary reporter ion interference. These results were used to propose solutions to mitigate the limitations of multi-batch TMT analyses. We confirm that including a common reference line in every batch increases precision by facilitating normalization across the batches and we propose experimental designs that minimize the effect of cross population reporter ion interference.

## Abstract (precis)

Analysis of 24 TMT 10-plex batches revealed an inflation in missing values and reduced inter-batch accuracy as multiple TMT batches are integrated. Our data also highlights the incidence of false positives exemplified by Y chromosome peptides being detected in female channels. The Y chromosome peptides were then used to quantify the effects of coisolation and reporter ion interference on TMT quantification and to propose an experimental design that would minimise cross population reporter ion interference.

## Graphical Abstract

**Figure:**
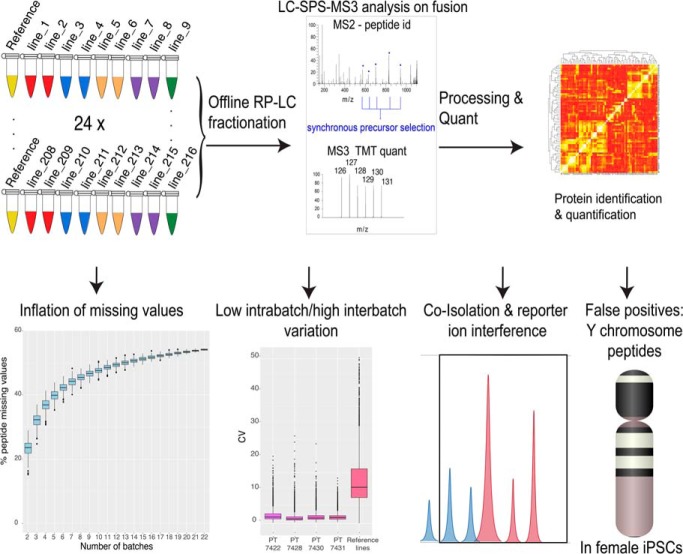

- Revealed inflation of missing values as multiple TMT 10-plex batches are integrated.
- Analyzed the impact of integrating multiple TMT 10-plex batches on the quantification accuracy of both high and low abundance proteins.
- Established reliable detection of false positives caused by coisolation and reporter ion interference, highlighted by the incidence of Y chromosome peptides in all female channels.
- Optimized new experimental design set-ups to minimize cross population reporter ion interference via insights into coisolation and reporter ion interference.

High-throughput, shotgun proteomics, using data dependent acquisition (DDA),^1^ now enables the comprehensive study of proteomes, allowing the identification of 10,000 or more proteins from cells and tissues (1–3). However, to achieve such deep proteome coverage using DDA, extensive prefractionation of extracts before mass spectrometry (MS) analysis is frequently required (1, 4). To evaluate statistically the significance of the resulting data, a minimum of 3 replicates for each sample/condition is also necessary (5, 6). The data acquisition time involved is increased still further for experiments that analyze the multi-dimensional characteristics of the proteome; for example, studying differences in protein subcellular localization, turnover rates, post-translational modifications (PTMs) and protein-protein interactions (7–9).

To cope with the challenges of large-scale proteomics analyses, strategies have been developed to allow multiple samples to be analyzed in parallel, through multiplexing isotopically tagged peptides (10, 11). The most widely used MS multiplexing methods, TMT (12) and iTRAQ (13), use isobaric tags for simultaneous peptide identification and quantification. TMT has increased in popularity and is now widely used (14, 15). This reflects the ability of multiplexed TMT to increase sample throughput in proteomics studies and reduce the “missing values” problem that arises from the stochastic sampling inherent in DDA proteomics (16, 17). Thus, within a single multiplexed TMT batch, the number of missing values at the protein level is low, frequently <2% (14). Further, the precision of the quantification within a multiplexed TMT batch is high (18). However, it is less clear how well multiplexed TMT performs for very large-scale analyses, involving numerous TMT batches.

In this manuscript, we use a proteomic data set of human iPSC cells, involving 24 separate 10-plex TMT batches (19). We compare the quantitation of data both within and between 10-plex batches and focus our analysis on 4 main issues: (1) missing values, (2) accuracy of quantification, (3) false positives and (4) the effect of both reporter ion interference (RII) and coisolation interference (CII).

We show there is an inflationary effect on missing values as data from multiple batches are integrated both at the protein and peptide level. We evaluated reproducibility both by studying the coefficient of variation (CV) within each 10-plex TMT batch, and by comparing a reference line (technical replicates of the iPSC line “bubh_3”) that were common to every batch. Furthermore, the incidence of false positives was studied by using Y chromosome peptides as an internal control. The iPSC lines quantified in this data set were derived from 163 different donors including both male and female, hence the peptides mapped to the Y chromosome should be absent from female lines. Nonetheless, we confirm that these Y chromosome-specific peptides were consistently detected in the female channels of all TMT batches. Finally, by using these Y chromosome peptides, we quantified the effect of ion coisolation and reporter ion interference upon TMT quantification accuracy.

## EXPERIMENTAL PROCEDURES

### 

#### 

##### Experimental Design and Statistics Rationale

The study consists of 240 iPSC replicates, 217 biological replicates and 24 technical replicates, derived from 163 different donors. The study comprises twenty-four 10-plex TMT batches. Each batch consisted of 1 common reference line (technical replicates of iPSC cell line “bubh_3”) and 9 different iPSC cell lines. The technical replicates were used for the data normalization strategy described below. Out of the 240 replicates analyzed, 142 were derived from female donors and from 98 male donors.

##### TMT Sample Preparation

For protein extraction, iPSC cell pellets were washed with ice cold PBS and redissolved immediately in 200 μl of lysis buffer (8 m urea in 100 mm triethyl ammonium bicarbonate (TEAB)) and mixed at room temperature for 15 min. Cellular DNA was sheared using ultrasonication (6 × 20 s on ice). The proteins were reduced using tris-carboxyethylphosphine TCEP (25 mm) for 30 min at room temperature, then alkylated in the dark for 30 min using iodoacetamide (50 mm). Total protein was quantified using the EZQ assay (Thermo Fisher Scientific, Waltham, MA). For the first digestion with mass spectrometry grade lysyl endopeptidase, Lys-C (Wako, Japan), the lysates were diluted 4-fold with 100 mm TEAB then further diluted 2.5-fold before a second digestion with trypsin. Lys-C and trypsin were used at an enzyme to substrate ratio of 1:50 (w/w). The digestions were carried out overnight at 37 °C, then stopped by acidification with trifluoroacetic acid (TFA) to a final concentration of 1% (v:v). Peptides were desalted using C18 Sep-Pak cartridges (Waters, Millford, MA) following manufacturer's instructions.

For tandem mass tag (TMT)-based quantification, the dried peptides were re-dissolved in 100 mm TEAB (50 μl) and their concentration was measured using a fluorescent assay (CBQCA, Thermo Fisher Scientific). For each 10-plex TMT batch 100 μg of peptides from each cell line to be compared, in 100 μl of TEAB, were labeled with a different TMT tag (20 μg/ml in 40 μl acetonitrile) (Thermo Fisher Scientific), for 2 h at room temperature. After incubation, the labeling reaction was quenched using 8 μl of 5% hydroxylamine (Thermo Fisher Scientific) for 30 min and the different cell lines/tags were mixed and dried *in vacuo*.

The TMT samples were fractionated using off-line, high-pH reverse-phase (RP) chromatography: samples were loaded onto a 4.6 × 250 mm Xbridge BEH130 C18 column with 3.5-μm particles (Waters). Using a Dionex bioRS system, the samples were separated using a 25-min multistep gradient of solvents A (10 mm formate at pH 9) and B (10 mm ammonium formate pH 9 in 80% acetonitrile), at a flow rate of 1 ml/min. Peptides were separated into 48 fractions, which were consolidated into 24 fractions. The fractions were subsequently dried and the peptides re-dissolved in 5% formic acid and analyzed by LC-MS/MS.

### TMT LC-MS/MS

#### 

##### TMT-based Analysis

Samples were analyzed using an Orbitrap Fusion Tribrid mass spectrometer (Thermo Fisher Scientific), equipped with a Dionex ultra-high-pressure liquid-chromatography system (RSLCnano). RPLC was performed using a Dionex RSLCnano HPLC (Thermo Fisher Scientific). Peptides were injected onto a 75 μm × 2 cm PepMap-C18 pre-column and resolved on a 75 μm × 50 cm RP- C18 EASY-Spray temperature-controlled integrated column-emitter (Thermo Fisher Scientific), using a four-hour multistep gradient from 5% B to 35% B with a constant flow rate of 200 nl/min. The mobile phases were: 2% ACN incorporating 0.1% FA (solvent A) and 80% ACN incorporating 0.1% FA (solvent B). The spray was initiated by applying 2.5 kV to the EASY-Spray emitter and the data were acquired under the control of Xcalibur software in a data-dependent mode using top speed and 4 s duration per cycle. The survey scan is acquired in the orbitrap covering the *m*/*z* range from 400 to 1,400 Thomson with a mass resolution of 120,000 and an automatic gain control (AGC) target of 2.0 × 10^5^ ions. The most intense ions were selected for fragmentation using CID in the ion trap with 30% CID collision energy and an isolation window of 1.6 Th. The AGC target was set to 1.0 × 10^4^ with a maximum injection time of 70 ms and a dynamic exclusion of 80 s, the scan rate was set to “Rapid.”

During the MS3 analysis for more accurate TMT quantifications, 5 fragment ions were coisolated using synchronous precursor selection with a window of 2 Th and further fragmented using HCD collision energy of 55%. The fragments were then analyzed in the orbitrap with a resolution of 60,000. The AGC target was set to 1.0 × 10^5^ and the maximum injection time was set to 105 ms.

##### Machine, Blanks, and Standards

All of the TMT batches were analyzed on the same Orbitrap Fusion MS instrument (Thermo Fisher Scientific). Between each individual TMT experiment, one blank was run, followed by analysis of a 15 peptide Retention Time Calibration (RTC) standard, to evaluate retention time drift. This was followed by analysis of an MCF10a total cell digest standard to evaluate peptide and protein identifications. The last step consisted of analysis of two blanks, one with an oscillating gradient and one with the gradient matching the samples to be run.

##### Identification and Quantification

The data from all twenty-four 10-plex TMT batches were batches were analyzed using Maxquant (20, 21) v. 1.6.3.3. The FDR threshold was set to 1% for each of the respective Peptide Spectrum Match (PSM) and Protein levels. The data was searched with the following parameters; type was set to Reporter ion MS3 with 10plex TMT, stable modification of carbamidomethyl (C), variable modifications, oxidation (M), acetylation (protein N terminus), deamidation (NQ), Glutamine to pyro-glutamate (N terminus), with a 2 missed tryptic cleavages threshold, reporter mass tolerance set to 0.03 ppm. Minimum peptide length was set to 7 amino acids. Proteins and peptides were identified using UniProt (SwissProt December 2018). The run parameters are accessible at ProteomeXchange (22) via the PRIDE repository (23), along with the full MaxQuant (20) quantification output (PXD010557).

##### Filtering

All proteins that were marked as “Reverse,” “Potential Contaminants,” or “Only identified by site” were discarded. The final subset comprised 9,640 proteins. Peptides marked as “Potential contaminants” or “Reverse” were also filtered from the analysis. The final peptide data set comprised 178,491 peptides.

##### Copy Number Generation

Protein copy numbers were calculated following the proteomic ruler approach (24). For protein, *p*, *uCN_b,c,p_* is the uncorrected protein copy number:

$$\begin{array}{c} uCN_{b,c,p}=protein\;MS3\;signal_{b,c,p}\times \frac{A}{M_{p}}\times \frac{6.85\times 10^{-12}}{\sum_{h\in b,c}histones\;MS3\;signal_{h}}\\ \text{for}\;\text{batch}\;b\in \left\{1,2,\ldots 24\right\}\;\text{and}\;\text{channel}\;c\in \left\{126C,127N,\ldots 131N\right\} \end{array}$$

where *A* is Avogadro's constant, *M_p_* is the molar mass of the protein *p*, protein MS3 signal is the protein MS3 intensity and histones MS3 signal is the MS3 intensity for all histones, *h*.

These uncorrected copy numbers, which will be referred to here as “raw copy numbers”, were used to study the coefficient of variation (CV). To control for technical variation between the 24 different 10-plex batches, a correction factor, *cf*, was applied to every protein, *p*, in every batch, *b*, to adjust the protein copy numbers.

$$\begin{array}{c} cf_{b,p}=\frac{uCN_{b,126C,p}}{\sum_{b}uCN_{b,126C,p}/24}\\ \text{for}\;b\in \left\{1,2,\ldots 24\right\} \end{array}$$

where *uCN*_*b*,126*C*,*p*_ is the protein copy number derived from reporter channel 126C (the reference channel). The normalized copy number, *normCN*, is calculated for protein, *p* in all batches, *b*, and all channels *c*:

$$\begin{array}{c} normCN_{b,c,p}=\frac{uCN_{b,c,p}}{cf_{b,p}}\\ \text{for}\;\text{batch}\;b\in \left\{1,2,\ldots 24\right\}\;\text{and}\;\text{channel}\;c\in \left\{126C,127N,\ldots ,131N\right\} \end{array}$$

##### Missing Value Calculations

First, to estimate missing values within this DDA analysis, a list of unique proteins/peptides that were detected with at least 1 reporter intensity greater than zero were calculated for each batch. To determine the number of missing values within each 10-plex TMT batch, the number of unique proteins/peptides per reporter channel was compared with the number of unique proteins/peptides identified within the batch. This approach was applied to generate the missing value calculations for each of the 24 individual 10-plex TMT batches.

To assess the effect of integrating multiple TMT batches, random sampling was performed to estimate how missing values are affected by a progressive increase in the number of 10-plex TMT batches analyzed. This was performed in an incremental fashion starting from 2 and finishing with 22 batches (PT6388 was not used for this analysis), with 500 iterations per level. At the first level 2 batches would be selected at random with no replacement, and at the last level 22 batches would be selected at random, again with no replacement. This was performed with the R function “sample()” part of the base R-core package.

At each level a new list of proteins/peptides detected with at least 1 reporter ion intensity greater than zero within any of the integrated TMT batches was calculated, and the number of proteins/peptides with intensity greater than 0 per reporter channel was evaluated against the new list.

##### Coefficient of Variation

The coefficient of variation (CV) in protein abundance levels was calculated using the log10 transformed protein copy numbers.

$$CV=\frac{S}{X}\times 100$$

For each protein the CV is equal to the copy number standard deviation (S) divided by the mean copy number (X) times 100. The protein CV within each 10-plex TMT batch was calculated for all 10 cell lines within the same batch, using all proteins detected in every reporter channel. The reference line CV was calculated using proteins that were detected in the TMT^10^-126C (reference line) channel across all of the 24 10-plex TMT batches.

##### Correlation Clustering

For each 10-plex TMT batch, a concordance correlation value was calculated for all cell lines within the same batch. The calculations were performed using “correlation()” function from the R package “agricolae” version 1.2.8.

The same process was applied to calculate the concordance correlation values for the reference lines, *i.e.* using reporter channel 126C in all TMT batches.

##### Peptide Intensity Normalization

The replicate normalized intensity, *rni*, was calculated per peptide, *q*:

$$\begin{array}{c} rni_{q}=\log_{10}\left(\frac{peptide\;MS3\;signal_{b,c,q}}{median\left(I_{b,c}\right)}\right)\\ I_{b,c}=\left\{peptide\;MS3\;signal_{b,c,q}:\forall q\right\}\;\text{given}\;\text{batch},\;b\;\text{and}\;\text{channel},\;c\\ \text{for}\;\text{batch}\;b\in \left\{1,2,\ldots 24\right\}\;\text{and}\;\text{channel}\;c\in \left\{126C,127N,\ldots ,131N\right\} \end{array}$$

The median normalized intensity, *mni*, for peptide, *q*, is the median of all batches, *b*, and channels, *c*:

$$\begin{array}{c} mni_{q}=median\left(rni_{b,c,q}\right)\\ \text{for}\;\text{all}\;\text{batches}\;b\in \left\{1,2,\ldots 24\right\}\;\text{and}\;\text{channels}\;c\in \left\{126C,127N,\ldots ,131N\right\} \end{array}$$

The *global median* is the median of *mni* for all peptides, *q*:

$$global\;median=median\left(mni_{q}\right)$$

##### Reporter Ion Interference Classification

The reporter ion interference (RII) targets are based on a typical product data sheet for 10-plex TMT Label Reagents from ThermoFisher Scientific, as summarized in Table I below:

**Table I:** Reporter ion interference classification for all TMT batches, specifying the reporter mass tag, the reporter channel within the MaxQuant output and the target channels for primary (+1 Da) and secondary (−1 Da) reporter ion interference

Mass tag	Reporter channel	−1Da (secondary RII)	+1Da (primary RII)
TMT^10^-126	1	–	127C
TMT^10^-127N	2	–	128N
TMT^10^-127C	3	126	128C
TMT^10^-128N	4	127N	129N
TMT^10^-128C	5	127C	129C
TMT^10^-129N	6	128N	130N
TMT^10^-129C	7	128C	130C
TMT^10^-130N	8	129N	131
TMT^10^-130C	9	129C	–
TMT^10^-131	10	130N	–

##### Analysis of Reporter Ion Interference

To study the effect of reporter ion interference across different TMT channels, we selected a subset of 69 peptides that were specific to the following list of protein coding genes uniquely located on the Y chromosome; “CDY1,” “CDY2A,” “DDX3Y,” “EIF1AY,” “KDM5D,” “NLGN4Y,” “PCDH11Y,” “RPS4Y1,” “TBL1Y,” “USP9Y,” and “UTY.”

This approach of using peptide values from Y chromosome specific genes depends upon there being a diverse mixture of male and female donor-derived iPSC lines in each 10-plex TMT batch. However, two of the 24 TMT batches comprised exclusively female donor-derived iPSCs, which had been shown not to have Y chromosome derived DNA in QC analyses (25). For these female donor-specific batches, any peptide assigned to Y chromosome specific genes was excluded from the analysis. An additional batch, PT6388, displayed an irregular behavior, and was hence also discarded from the posterior analysis. A final subset of 65 Y chromosome-specific peptides were used for this analysis (see supplemental data for list).

##### Peptide Male Versus Reporter Ion Interference Ratios

The peptide ratios comparing male channels *versus* female channels, *mpr*, subjected to different reporter ion interference conditions, *cond*, were calculated per 10-plex TMT batch, *b*, for peptide, *q*, using the replicate normalized intensities:

$$\begin{array}{c} mpr_{b,q}\frac{median\left(RNI_{b,male,q}\right)}{median\left(RNI_{b,cond,q}\right)}\\ RNI_{b,male,q}=\left\{rni_{b,c,q}:\forall male\;channels,c\right\},\\ RNI_{b,cond,q}=\left\{rni_{b,c,q}:\forall cond\;channels,c\right\},\\ \text{for}\;\text{b}\in \left\{1,2,\ldots 24\right\}\;\text{and}\;cond\in \left\{\text{primary}\;\text{RII},\text{secondary}\;\text{RII},\text{double}\;\text{RII},\text{no}\;\text{RII}\right\}. \end{array}$$

The box plot comparing male replicates to the different reporter ion interference conditions used these peptide batch ratios and was plotted using “ggplot2” version 3.0.0 (26).

##### Peptide Reporter Ion Interference Ratios

The peptide ratios, *npr*, comparing different reporter ion interference (RII) conditions, *cond*, in female channels to female channels with no reporter ion interference, *noRII*, were calculated within each 10-plex TMT batch, *b*, for peptide, *q*, using the replicate normalized intensities.

$$\begin{array}{c} npr_{b,q}\frac{median\left(RNI_{b,cond,q}\right)}{median\left(RNI_{b,noRII,q}\right)}\\ RNI_{b,cond,q}=\left\{rni_{b,c,q}:\forall cond\;channels,c\right\},\\ RNI_{b,noRII,q}=\left\{rni_{b,c,q}:\forall noRII\;channels,c\right\},\\ \text{for}\;b\in \left\{1,2,\ldots 24\right\}\;\text{and}\;cond\in \left\{\text{primary}\;\text{RII},\text{secondary}\;\text{RII},\text{double}\;\text{RII}\right\}. \end{array}$$

These results were stratified by the global median, where peptides with median normalized either intensity greater than or equal to the global median were considered 'High intensity' and those lower than the global median were considered 'Low intensity'. The box plot comparing different reporter ion interference conditions to the replicates not affected by reporter ion interference used these peptide batch ratios and was plotted using “ggplot2” version 3.0.0 (26).

## RESULTS

### 

#### 

##### Missing Values in TMT

A known advantage of using TMT is the low index of missing values that are present within a single TMT batch. Recent studies report as low as <1% missing values at the protein level (18), albeit data are usually not reported at the peptide level.

**Fig. 1.**
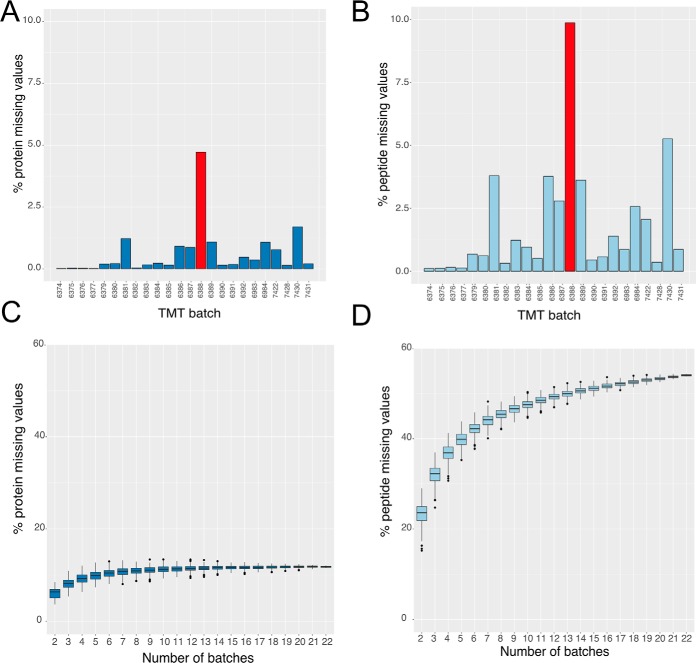
**Protein and peptide missing values:** *A*, Percentage of missing values for each TMT batch calculated at the protein level. *B*, Percentage of missing values for each TMT batch calculated at the peptide level. *C*, Box plot showing the results for protein missing values as a function of the number of 10-plex TMT batches (see methods). *D*, Box plot showing the results for peptide missing values as a function of the number of 10-plex TMT batches (see methods). For both *C* and *D* the lower and upper hinges represent the 1st and 3rd quartiles. The upper whisker extends from the hinge to the largest value no further than 1.5 * IQR from the hinge, the lower whisker extends from the hinge to the smallest value at most 1.5 * IQR of the hinge.

We started by analyzing the iPSC 10-plex TMT data for the number of missing values at the protein level within each TMT batch (Fig. 1*A*). The preliminary results are consistent with previous reports, *i.e.* 92% of the 24 different 10-plex TMT batches show <1% missing values at the protein level, with only 1 outlier with missing protein values >1.5%. This was experiment PT6388, which is highlighted in red (Fig. 1*A* and 1*B*). Further, when we analyze the data at the peptide level, there is very close agreement to the protein data, with 92% of the 24 different 10-plex TMT batches having <5% missing peptide values, however the outlier batch has an exacerbated effect and displays 9% missing values at the peptide level. We therefore excluded PT6388 from the rest of the analysis.

These previous results do not address the effect of integrating data from multiple, independent 10-plex TMT batches into a single analysis. To study the effect of data integration, we increased the number of batches selected, from 2 to 22 and recalculated the number of missing values that were present (Fig. 1*C* and 1*D*; see methods). At the protein level, the median number of missing values increases from 0.19% with one 10-plex TMT batch, to 6.35% when data from a second 10-plex TMT batch were integrated (Fig. 1*C*). When we integrate data from 5 different 10-plex TMT batches, the median number of missing values at the protein level escalated to ∼10%.

This situation was exacerbated when the analysis was performed at the peptide level (Fig. 1*D*). When integrating data from just two 10-plex TMT batches, the median number of missing peptide values was >23%. Even more striking, it only required integrating data from 5 different 10-plex TMT batches to produce ∼40% missing values at the peptide level. The data suggest peptides are not reproducibly detected among batches, but is it only low abundance peptides?

**Fig. 2.**
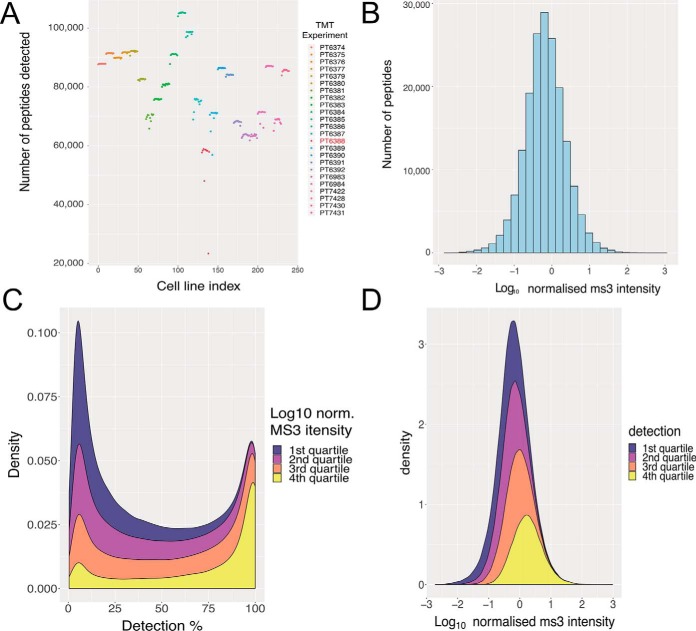
**Peptide identifications and intensities:** *A*, Number of peptides identified with MS3 intensity greater than zero in all TMT channels, colored by TMT batch. *B*, Histogram of the median normalized peptide intensity (see methods). *C*, Stacked density plot showing peptides grouped by median normalized peptide intensity quartiles and their percentage of detection across all TMT channels. *D*, Stacked density plot showing quartiles of identification rates for each peptide and their corresponding log_10_ normalized MS3 intensity.

Based upon these results, we decided to perform a more in-depth analysis on the inflation rate of peptide missing values. We observed that the number of peptides identified within each 10-plex TMT batch is relatively constant (Fig. 2*A*), but quite variable across different batches. The median number of peptides identified per batch was 84,046 with a standard deviation of 11,354. To further analyze these peptide level data, we first median-normalized the MS3 intensities for all peptides in all cell lines (see methods). The log_10_ median normalized MS3 intensities spanned 6 orders of magnitude (see methods; Fig. 2*B*).

We next analyzed the peptide data set by quartiles, based on the log_10_ median normalized intensity values (Fig. 2*C*). The first quartile represented the 25% least abundant peptides and the fourth quartile the 25% most abundant peptides. There are only 11 peptides within the first quartile that are detected in all TMT channels and only 603 peptides that are seen in > 90% of the TMT channels. As DDA selects the n most abundant ions reaching the mass-spectrometer during a MS1 scan (27) (where *n* typically is 10–30), this bias is predictable. However, when we analyze the results from the fourth quartile (25% most abundant peptides), we see that 26% of these peptides are still only detected in <50% of the TMT channels. These high intensity peptides that are detected in less than half of all channels have a median normalized intensity of 0.32, representing the 84th percentile of abundance. Furthermore, only 24% of the peptides from the 4th quartile were detected in all the TMT channels. In total there are 12,140 peptides that were detected in all TMT channels, regardless of intensity classification, which represents 6.81% of all peptides.

Next, we analyzed the data by comparing the identification quartiles, organized by the percentage of TMT channels in which they were detected (Fig. 2*D*). The first quartile represented the 25% of peptides that were detected least frequently, *i.e.* in less than 29 TMT channels (see supplemental data). Of these peptides >29% had a median normalized intensity higher than the global median (see methods; −0.184), highlighting that even relatively abundant peptides are not identified consistently. Overall, ∼50% of peptides are detected in <40% of all TMT channels.

##### Variation Between 10-plex TMT Batches

Multiple studies have documented TMT as a method producing precise quantitation, in some cases having a coefficient of variation (CV) ∼3x lower than comparable label free data (18). Most of these studies have focused on analyzing quantitative precision within a single TMT batch, and do not explore the effect of integrating data from multiple TMT batches into one analysis. However, projects involving large scale proteomic analyses of multiple cell lines and/or conditions, need to employ multiple TMT batches in a single experiment (10).

We calculated protein copy numbers for 230 iPSC replicates, which included 208 biological replicates and 23 technical replicates of a control iPSC line (bubh_3), across 23 separate 10-plex TMT batches (PT6388 is once again removed from the analysis). We then proceeded to calculate Lin's concordance correlation coefficient, which measures the agreement between two variables and is proposed to evaluate reproducibility (28). This was performed for every iPSC line within each TMT 10-plex batch, and for all the technical replicates of the control line, channel TMT^10^ 126, across the 23 10-plex TMT batches.

The concordance correlation coefficient within each 10-plex TMT batch is very high, (median value 98% concordance), highlighting the precision of the quantitation within each single batch. However, when the same calculation is applied to the technical replicates of the control iPSC line across 23 respective batches, the median concordance coefficient drops to 81%.

**Fig. 3.**
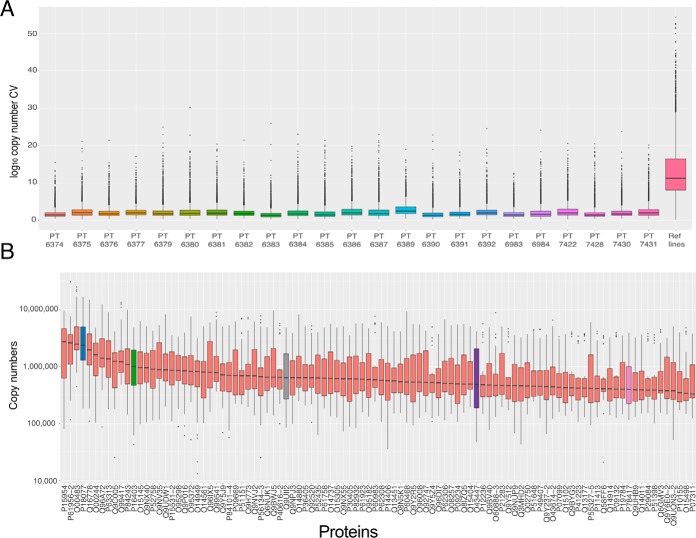
**Variation:** *A*, Box plots showing the protein copy number coefficient of variation for all proteins detected in each 10-plex TMT batch as well as all proteins detected in all the reference line replicates (TMT channel 126C in all batches). *B*, Box plots showing the protein copy numbers of the 100 most abundant proteins with a coefficient of variation greater than or equal to 7.5 across all reference line replicates (TMT channel 126C in all batches). For both boxplots the lower and upper hinges represent the 1st and 3rd quartiles. The upper whisker extends from the hinge to the largest value no further than 1.5 * IQR from the hinge, the lower whisker extends from the hinge to the smallest value at most 1.5 * IQR of the hinge.

To explore this situation further, we calculated the CV for the log_10_ transformed protein copy numbers (29) (see Methods), both within each 10-plex TMT batch, and across 23 controls (Fig. 3*A*). When we calculated the protein CV exclusively within each 10-plex TMT batch, the median was 1.72 with all 10-plexes showing a median protein CV <2.5. Accordingly, the data show that for every batch, proteins with a CV >7.5 were considered outliers (Fig. 3*A*). These data show high precision of quantitation within each individual multiplexed experiment.

Similarly, to evaluate accuracy we calculated the CV for all technical replicates of the reference iPS cell line, bubh_3, which were analyzed in channel TMT^10^ 126 in every 10-plex TMT batch. The median CV of all the proteins detected in the technical replicates, was 11.03, that is, 6.4-fold higher than the median within-batch CV. The CV of over 80% of all proteins in the technical replicates would be considered outliers in all the within-batch 10-plex TMT analyses.

It is commonly assumed in proteomics studies that variation predominantly affects low intensity proteins and peptides. We decided to test this assumption and focused on the extremely abundant proteins. We focused the analysis on the 23 reference-line replicates, and selected the top 100 most abundant proteins where the CV was greater than 7.5 and created box plots based on their copy numbers across all reference line data (Fig. 3*B*).

We chose to highlight 5 cases; 60S ribosomal protein L35a (RPL35A; highlighted in blue), Histone H1.2 (HIST1H1C; highlighted in green), ATPase inhibitor, mitochondrial (ATP5IF1; highlighted in gray), Peptydyl-prolyl cis-trans isomerase H (PPIH; highlighted in purple) and Glutathione S-transferase omega-1 (GSTO1; highlighted in pink). RPL35A ranges in expression from ∼16,786,000 to ∼185,000 copies and was identified with 10 unique + razor peptides (URP). HIST1H1C ranges from ∼4,847,000 to ∼43,000 copies and was identified with 6 URP, ATP5IF1 from ∼4,825,000 to ∼84,000 copies and was identified with 6 URP, PPIH from ∼4,180,000 to ∼45,000 copies and was identified with 14 URP and GSTO1 from ∼3,920,000 to ∼115,000 and was identified with 16 URP. All of these 5 proteins are highly abundant, with a median copy number > 500,000 within the 23 technical replicates of the reference line, have been identified with >6 URP and yet they vary drastically between the different multiplexed experiments, highlighting that the variation is not limited to low abundance proteins.

We also note that although the majority of iPSC lines in this study come from healthy donors, some of these TMT batches, for example, PT6390, contain mixtures of iPSC lines derived from both healthy donors and donors with rare genetic diseases, including “Usher syndrome”, “Monogenic Diabetes,” and “Bardet-Biedl syndrome.” Nonetheless, the median protein CV within PT6390 is still ∼10 fold lower than the CV obtained from analyzing the 23 technical replicates of bubh_3, indicating that TMT batch effects have a bigger influence on the proteomics data than a healthy *versus* diseased physiology.

Our results highlight that although multiplex TMT is a useful and precise methodology for quantitative proteomics, it is important to be aware also of its potential limitations, particularly when analyzing data from multiple TMT batches. It is important to note that copy numbers already provide a layer of normalization (24), however it proves insufficient to deal with batch variation. These findings underline that when conducting very large-scale proteomics analyses across multiple TMT batches, it is essential to be aware of the potential for batch variation to affect data quality. To reduce the batch effects several methods have been proposed, from protein expression inference (30), to a standard reference line (31). Here we used the technical replicates of a reference iPSC line as an internal reference standard to control for variation between batches (see methods). Using this normalization method provided a median CV of 2.96% across all cell lines and technical replicates, making the results closer to the metrics obtained for each within-batch analysis.

##### False Positives

The iPSC data set (19, 25) provided us with an excellent opportunity to study the incidence of false positives within a multi-plex TMT batch. The study used iPSC lines derived from both male and female donors within twenty-two of the twenty-four 10-plex TMT batches analyzed here. Because only the lines from male donors should include proteins encoded by genes exclusively on the Y chromosome, this provided effectively a set of endogenous “spike-in” peptides, which we could use to monitor the presence of false positives as well as exploring the effects of reporter ion interference (RII) between TMT channels and coisolation interference (CII).

The data set detected 11 proteins that were mapped specifically to the Y chromosome. Correspondingly, all unique peptides derived from these proteins should only be present in the TMT channels with male cell lines and, in theory, should be absent in the TMT channels with female cell lines. To avoid mismatches arising from shared peptides, we focused our analysis on a subset of 69 peptides that mapped uniquely to the following Y chromosome specific genes; “CDY1,: “CDY2A,: “DDX3Y,: “EIF1AY,: “KDM5D,: “NLGN4Y,: “PCDH11Y,: “RPS4Y1,: “TBL1Y,: “USP9Y,: “UTY.” Additionally, because two of the 10-plex TMT batches analyzed (PT6384 and PT7422) had only female cell lines, any potential Y chromosome-specific peptides that were detected in these batches were treated as wrongly annotated and discarded from further analysis. Furthermore, batch PT6388 was also considered an outlier and not included for further analysis. As a result, we focused on 65 unique, Y chromosome encoded peptides that were used as “male-specific” spike-in references for the analysis of false positives within the previously mentioned 21 TMT batches.

**Fig. 4.**
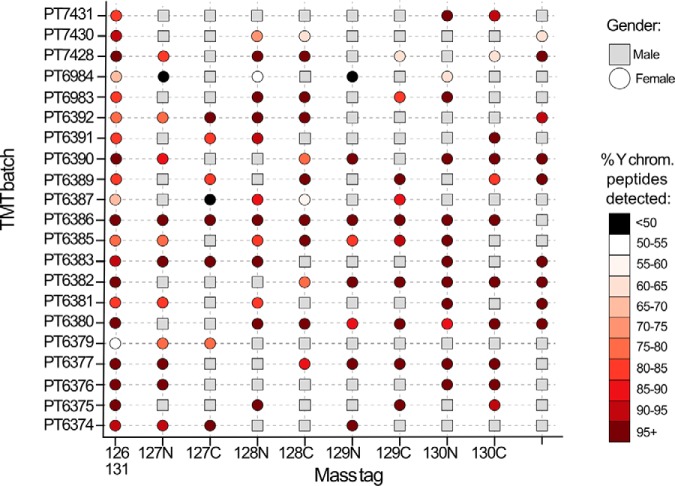
**Y chromosome peptides in female channels:** Scatter plot showing the gender/incidence of false positives across 21 TMT batches and their reporter ion mass tags. Male cell lines are shown as a gray square, female cell lines are represented by a circle. The female lines (circles) are shaded to indicate the percentage of Y chromosome specific peptides that were detected in their channel within each TMT batch.

We evaluated false positives by exploring how frequently the respective female TMT channels were quantifying signal from Y chromosome-specific peptides (Fig. 4). Surprisingly, this showed that in all twenty-one 10-plex TMT batches considered here and in all reporter channels containing a female cell line, a minimum of over 40% of the Y chromosome-specific peptides identified within the batch also had signal in the female channels. Remarkably, across all these batches, a median of 89% of Y chromosome-specific peptides quantified in each batch were quantified in TMT channels that contained female cell lines. The Y chromosome peptides should not be present in any female line, hence the level of detection mentioned earlier is unexpected. We infer that the appearance of signal for Y chromosome-specific peptides in the channels containing female cell lines likely results from a combination of coisolation and reporter ion interference.

##### Reporter Ion Interference and Coisolation Interference

**Fig. 5.**
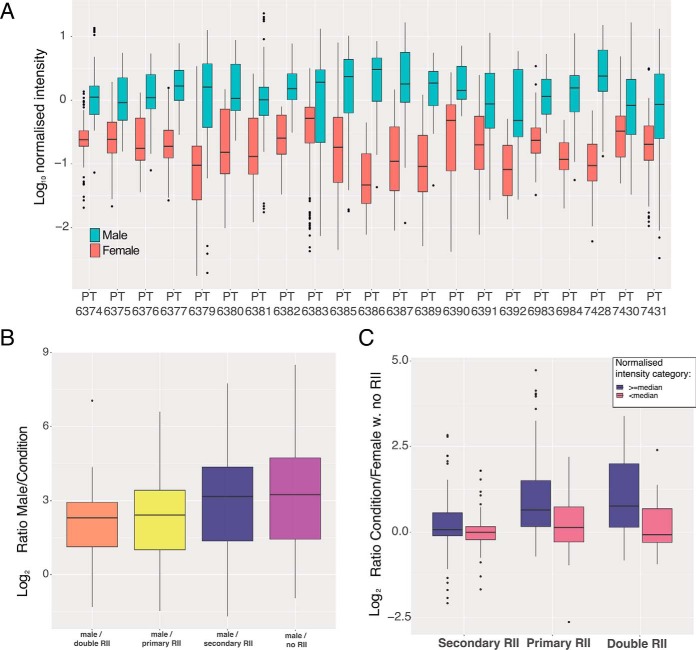
**TMT channel leakage analysis:** *A*, Box plot showing the median normalized intensity of Y chromosome specific peptides for both female and male cell lines across 21 TMT batches. *B*, Box plot of ratios for Y chromosome specific peptides, comparing male channels *versus* female channels affected by different reporter ion interference type. *C*, Box plot of ratios for Y chromosome specific peptides, stratified by the median normalized intensity, comparing female channels affected by different types of reporter ion interference *versus* female channels not subjected to reporter ion interference. For all 3 boxplots the lower and upper hinges represent the 1st and 3rd quartiles. The upper whisker extends from the hinge to the largest value no further than 1.5 * IQR from the hinge, the lower whisker extends from the hinge to the smallest value at most 1.5 * IQR of the hinge.

Reporter ion interference, also known as cross-label isotopic impurity, can arise from manufacture level impurities and experimental error (32). Coisolation interference is the effect caused by multiple labeled peptides being selected within the isolation window (33). To study both conditions we focused on the previously mentioned Y chromosome-specific peptides, as these should only be present in the male channels and absent in the female channels, therefore any signal detected in female channels should be artificial. We used the Y chromosome peptides to evaluate the difference in replicate normalized peptide intensities (see Methods) between male and female lines, across all the twenty-one 10-plex TMT batches (Fig. 5*A*).

The results of the analysis revealed significant variation between TMT batches. For example, some batches, such as PT6379 and PT6386, have 17 and 65-fold difference in intensity between male and female channels, simplifying the detection of false positives because of coisolation interference. However, other batches, *e.g.* PT7430 and PT6391, only show a 2.5 and 4.4-fold difference, respectively making the detection of the false positives problematic. We note both previously mentioned batches display low intensity peptides and hence low signal-to-noise ratios, making female channels more vulnerable to coisolation interference.

To evaluate coisolation ion interference, we selected female channels with no primary or secondary reporter ion interference (no cross-label isotopic impurity; see methods), as likely examples of coisolation interference (34). Peptides in male channels show a median of 9.43-fold higher replicate normalized intensity (see methods) compared with female channels not affected by reporter ion interference. However, the effects vary depending on the peptide intensity thresholds. High intensity peptides, where the median normalized peptide intensity across male lines was greater than or equal to the global median (-0.184; see methods), displayed 12.8-fold higher intensities. Low intensity peptides, where the median normalized peptide intensity of male lines was lower than the global median, only displayed 2.14-fold increased intensity, revealing higher vulnerability to coisolation interference.

We also examined the potential effects of reporter ion interference (RII). For this analysis, we calculated a peptide specific ratio for each condition (see methods), firstly comparing the male channels *versus* the different types of reporter ion interference present in female channels. 'Primary RII' occurs when a male channel affects by isotopic impurity the +1Da female channel of the same isotope (male 126C to female 127C). 'Secondary RII' occurs when a male channel affects by isotopic impurity the −1Da female channel of the same isotope (male 127C to female 126C). 'Double RII' occurs when a female channel is affected by both 'primary RII' and 'secondary RII' from male channels. Channels not affected by either primary, or secondary RII were labeled as “no RII.” The ratios comparing males to the previous conditions were used to generate the box plot (Fig 5*B*).

The male lines were a median of 9.43-fold higher than female channels not affected by reporter ion interference (“no RII”), but only 4.9-fold higher than female channels subjected to primary and secondary reporter ion interference (“double RII”). The smallest effect was caused by “secondary RII” where the male lines were 8.94 fold higher than the female lines, suggesting that “primary RII” is the main source of isotopic impurities and explaining why we see little difference between the “primary RII” and “double RII” conditions. We note that for both “primary RII” and “double RII” the false positives are within the 8-fold increase/decrease range for *bona fide* changes in protein/peptide expression levels often detected within proteomic data sets (35, 36).

To quantify the differences between primary and secondary reporter ion interference across peptide abundance categories, we now compared female channels affected by reporter ion interference (“primary RII,” “secondary RII,” and “double RII”) to female channels not affected by reporter ion interference (Fig. 5*C*; see Methods). We also stratified this analysis by the median normalized peptide intensity, with high intensity values being either greater than or equal the global median, and low intensity being lower than the global median. Within the analysis we confirmed that the smallest effect was caused by “secondary RII”; for high intensity Y chromosome-specific peptides it displayed only a 1.05-fold increase compared with the channels not affected by reporter ion interference and virtually no change in low abundance peptides (Fig 5*C*). The “primary RII” displayed a more pronounced effect, with a median increase of 1.57-fold in the high intensity peptides and 1.10-fold in the low intensity ones. The combination of primary and secondary RII produced a median of 1.7-fold increase in the high intensity peptides and a small reduction in the median for the low intensity peptides.

These results suggest low intensity peptides are mostly affected by coisolation interference, as reporter ion interference has little to no effect on their ratios, but that reporter ion interference can have profound effect in quantification of high intensity peptides. This provides important practical information that aids the design of TMT experiment to help minimize the potential effects on data quantification of cross condition/population reporter ion interference.

##### Optimizing the Experimental Design

For all studies based on more than a single multi-plex TMT batch, we advocate at least one relevant internal reference sample should be included in each batch and assigned to either channel 126C, or 127N. These channels avoid “primary RII,” the main cause of isotopic impurities, and are only affected by “secondary RII,” which only causes a median increase of 2.2% in intensity. In contrast, placing the reference line at channel 131N, or 131C increases the impact of reporter ion interference by exposing them to “primary RII.”

**Fig. 6.**
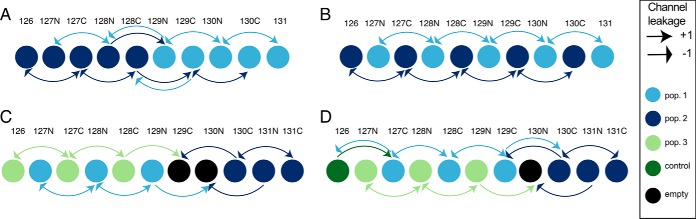
**TMT experimental design from reporter ion interference analysis:** *A*, 5–5 grouped layout for a 10-plex TMT batch with 2 populations and 5 replicates each. Two channels are being affected by cross population primary and secondary reporter ion interference. *B*, optimal layout for a 10-plex TMT batch with 2 populations and 5 replicates each, with no cross population primary or secondary reporter ion interference. *C*, optimal 11-plex configuration for 3 populations with 3 replicates each. By leaving two empty channels, it eliminates cross population reporter ion interference. *D*, optimal 11-plex configuration for 3 populations with 3 replicates each, with one empty channel and one reference line channel. Only two channels suffer primary and secondary reporter ion interference.

Our results also show TMT experimental designs that can help to minimize the effects of primary and secondary reporter ion interference between the different populations/conditions. For example, in a 10-plex TMT study, when two conditions are being analyzed, each with 5 biological replicates, a 5–5 grouped layout would cause multiple channels to be affected by cross population/condition reporter ion interference (Fig. 6*A*). The optimal design would involve alternating the two conditions across the 10 channels (Fig. 6*B*).

If a control is included, or for studies analyzing 3 different conditions in triplicate, (*e.g.* three time points, or a control and two different perturbations, etc.), we recommend using TMT 11-plex as all 10-plex TMT setups involve cross population/condition reporter ion interference. An 11-plex TMT set up enables a design without reporter ion interference between the 3 conditions/populations but requires two empty channels at 129C and 130N to achieve this (Fig. 6*C*). If a control channel is included, as advocated, then it should be placed in channel 126C, while locating the empty channel to position TMT^11^ 130N, between the alternating experimental conditions and the final replicates of the 3rd condition (Fig. 6*D*). All the suggested setups aim to reduce cross condition/population reporter ion interference, thereby avoiding decreases in quantification accuracy by isotopic impurities.

## DISCUSSION

Quantitative proteomic analysis using TMT labeling has become one of the most popular DDA methods currently used, thanks to its multiplexing capabilities, scalability, low missing values index and precision when a single multiplexed batch is analyzed. However, when large studies that require the use of multiple parallel TMT batches are performed (37, 38), the situation becomes more complicated. Here, we have used the analysis of data integrated from over 20, 10-plex TMT batches to investigate accuracy, missing values, false positives, coisolation interference, reporter ion interference and experimental design within very large-scale proteomics experiments. We have focused on a model data set derived from the analysis of human iPS cell lines, derived from both male and female donors (25).

The resulting data confirms that a single batch of multi-plex TMT experiments minimizes the typical missing values issue associated in proteomics with data dependent acquisition (DDA), both at the protein and peptide levels. However, this situation changes as data from two or more separate multi-plex TMT batches are integrated. As multiple batches are combined the missing values index inflates rapidly. This effect is particularly striking at the peptide level, where integrating data from only two different batches causes the missing values to increase from <2% to ∼24%. Even though the inflation rate at the protein level is lower, the integration of the second batch pushes missing protein values from <0.5% to >6%. This inflationary effect can decrease the accuracy of results derived from large-scale experiments that compare data generated from multiple TMT batches. One potential solution would be to use MS2-based TMT quantitation, as this has been reported to produce more total peptide identifications (39), however there is no guarantee this will detect peptides/proteins more reproducibly across batches. Furthermore, MS2-based TMT quantitation will intensify the disruptive effect of the coisolation interference.

Although single TMT batches can provide remarkably precise results within the multiplexed experiment, we found that this is often not reproducible across multiple batches. To study reproducibility, we normalized the data using the proteomic ruler (24) and for every protein we calculated the coefficient of variation (CV), both for the 23 technical replicates of the reference line, and within each of 10-plex TMT batches. The median copy number CV of the technical replicates was ∼6.4-fold higher than data from different donors analyzed within the same 10-plex TMT batch.

This also underlines the importance of normalizing batch effects, in our case via a common control sample within each TMT batch which allows for objective data normalization to minimize the batch effects, as has been reported (40, 41). Copy numbers apply a layer of normalization, but our data highlight they are insufficient to address batch effects. We showed that by introducing at least one suitable control (reference line) within each TMT batch the batch effects can be normalized effectively. The challenge lies in identifying a suitable control that is truly representative for most proteins being compared within the experiment and in creating a control that is highly reproducible across all the TMT batches.

This study has also highlighted the issue of false positives, reporter ion interference and coisolation interference. The data set we selected provided an ideal set up to analyze these factors, as it contained iPSC lines derived from both male and female donors. Thus, by identifying a set of peptides uniquely mapped to the male-specific Y chromosome, these provided a convenient set of internal controls to monitor the expression of false positives. The data showed that even for a 10-plex TMT batch with only two male channels (PT6380), the remaining 8 female channels still quantified 97.5% of all the Y chromosome-specific peptides that were detected in that batch. This means there are false positives being consistently detected in the female channels within the multiplexed experiments, which suggests there are severe limitations when analyzing heterogenous populations within the same TMT batch. This issue we attribute mostly to coisolation interference and we note that the issue has been reduced, though not completely eliminated, with newer generation Orbitrap MS instruments, where the improved source has enhanced the signal to noise ratio (34). Furthermore, new isobaric tagging methods have been developed which claim to be coisolation free (42), however their multiplexing capability is currently limited to a 6-plex.

We used the previously mentioned Y chromosome peptides to study the different reporter ion interference (RII) conditions across 21 different 10-plex TMT batches with different number of male and female derived cell lines, as well as different channel combinations. The data highlighted the effects of primary (male channel isotopic contamination into a +1Da female channel) and secondary (male channel isotopic contamination into a −1Da female channel) reporter ion interference. Reporter channels affected by both primary and secondary reporter ion interference showed a median signal increase in high intensity peptides of 1.7-fold compared with channels not subjected to reporter ion interference. This was found to be primarily caused by “primary RII” as the data also showed that “secondary RII” had the smallest effect with only a median 1.02-fold increase compared with channels with no reporter ion interference. To best avoid the effects of reporter ion interference, we have used these data to propose optimized experimental set ups for assigning samples to specific channels that can either minimize, or eliminate (when possible), the effect of primary and secondary reporter ion interference between conditions/populations. Nonetheless, we highlight again that mixing significantly different populations within a TMT batch, for example iPSCs and terminally differentiated somatic cells, will introduce false positives within the data, as illustrated here by the Y chromosome-specific peptides detected within all female cell lines.

For such large-scale experiments it is also vital to have strict quality control (QC) procedures in place to evaluate and maintain a constant performance within the instrument/s. In our case one of the TMT batches (PT6388) revealed poor performance in the QC run (see supplemental Figures). The failed QC run was not detected until after the samples were run, producing poor results within that batch. We therefore recommend that to execute large-scale TMT a rigorous QC procedure should be set in place before the start of the experiment.

In conclusion, TMT is a valuable methodology for DDA analysis and its potential to increase scalability and produce precise quantitation have made it a justifiably popular approach for high-throughput proteomic studies. Here, we have provided an in-depth, practical evaluation of parameters affecting the generation of high-quality quantitative data from very large-scale TMT-based proteomics analyses, and we highlight some of the limitations which should be carefully considered when planning these experiments. We hope the resulting information will prove useful for improving experimental design and resulting data quality for many future proteomics projects.

## Data Availability

The raw files used for this analysis are accessible at ProteomeXchange (22) via the PRIDE repository (23) with the dataset identifier PXD010557, along with the full MaxQuant (20) output.

## Supplementary Material

Number of peptides identified MS2

Number of peptides per protein acros batches

Number of proteins identified

Sequence coverage acros batches

Mising value iterations

Protein copy numbers

TMT experiment/channel mapping

Y chromosome peptide summary

QC vs Mising values

QC vs Proteins MS2

## Footnotes

^1^ The abbreviations used are:

DDA: data dependent acquisition
PTM: post-translational modification
RII: reporter ion interference
CII: coisolation interference
TMT: tandem mass tags.
