# Supplementary material for: Multibatch TMT Reveals False Positives, Batch Effects and Missing Values
Source: Mol Cell Proteomics. 2019 Jul 22;18(10):1967–80. doi: 10.1074/mcp.RA119.001472 (PMC6773557; doi:10.1074/mcp.RA119.001472)
Supplement: Number of peptides identified MS2 [file 144550_2_supp_346176_psxjvp.pdf]

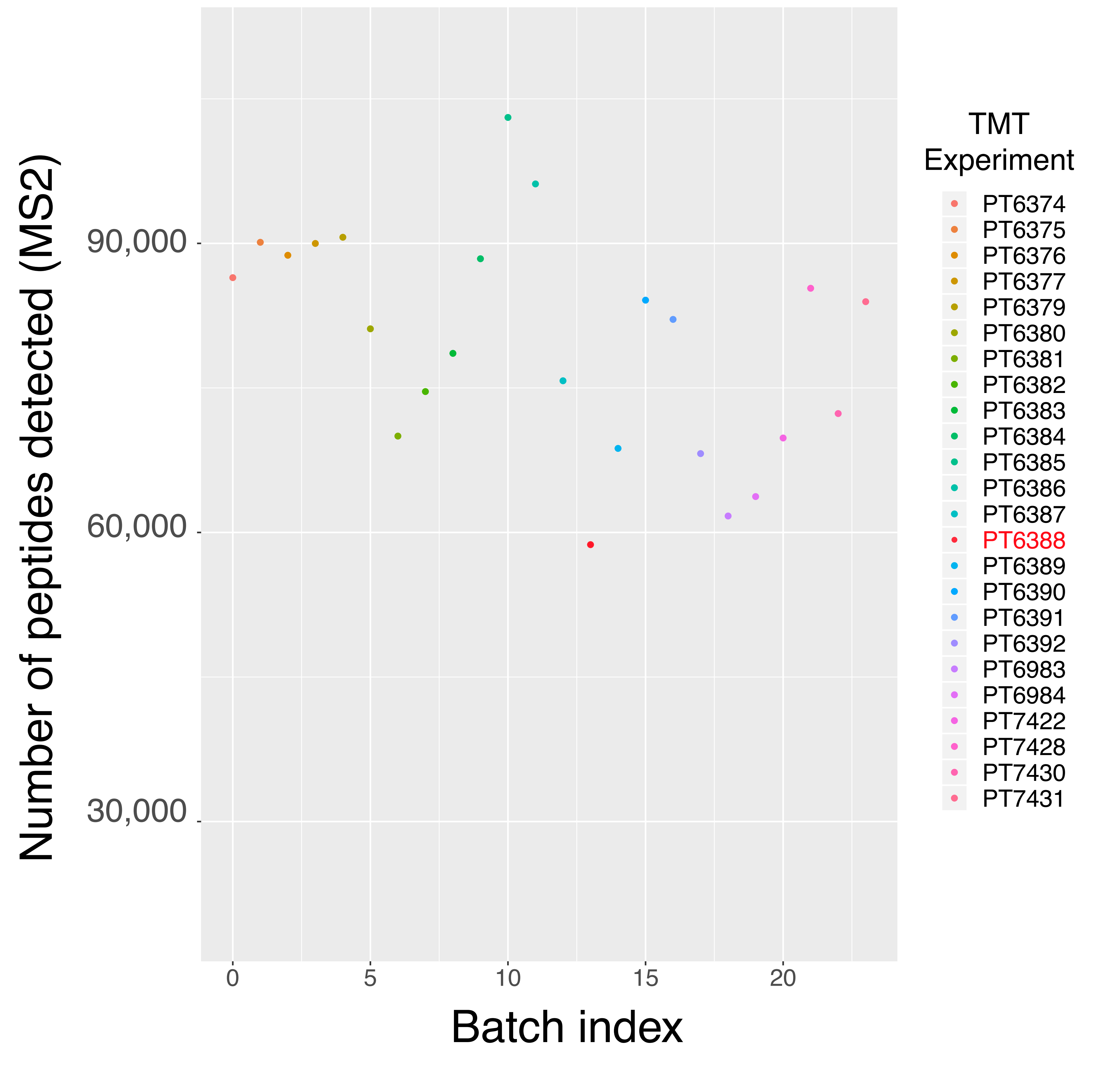

Number of peptides detected (MS2)
90,000
60,000
30,000
0
5
10
15
20
Batch index
TMT Experiment
PT6374
PT6375
PT6376
PT6377
PT6379
PT6380
PT6381
PT6382
PT6383
PT6384
PT6385
PT6386
PT6387
PT6388
PT6389
PT6390
PT6391
PT6392
PT6983
PT6984
PT7422
PT7428
PT7430
PT7431
