# Supplementary figures and images for: Multibatch TMT Reveals False Positives, Batch Effects and Missing Values
Source: Mol Cell Proteomics. 2019 Jul 22;18(10):1967–80. doi: 10.1074/mcp.RA119.001472 (PMC6773557; doi:10.1074/mcp.RA119.001472)

Number of peptides

256

32

4

pt 6374 pt 6375 pt 6376 pt 6377 pt 6379 pt 6380 pt 6381 pt 6382 pt 6383 pt 6384 pt 6385 pt 6386 pt 6387 pt 6388 pt 6389 pt 6390 pt 6391 pt 6392 pt 6983 pt 6984 pt 7422 pt 7428 pt 7430 pt 7431

TMT batch

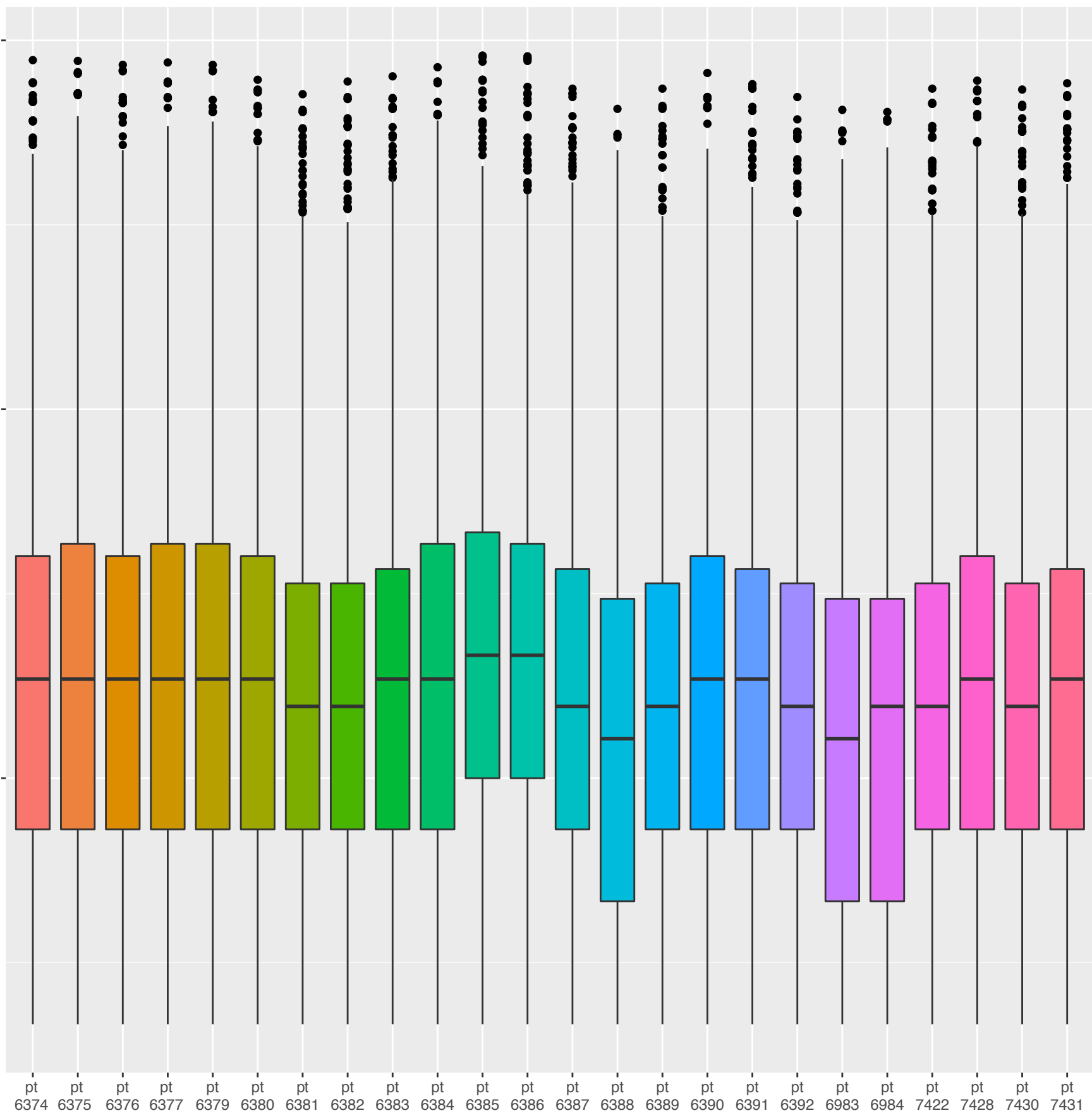

Supplement: Number of peptides per protein acros batches [file 144550_2_supp_346177_psxjxq.pdf]

% sequence coverage

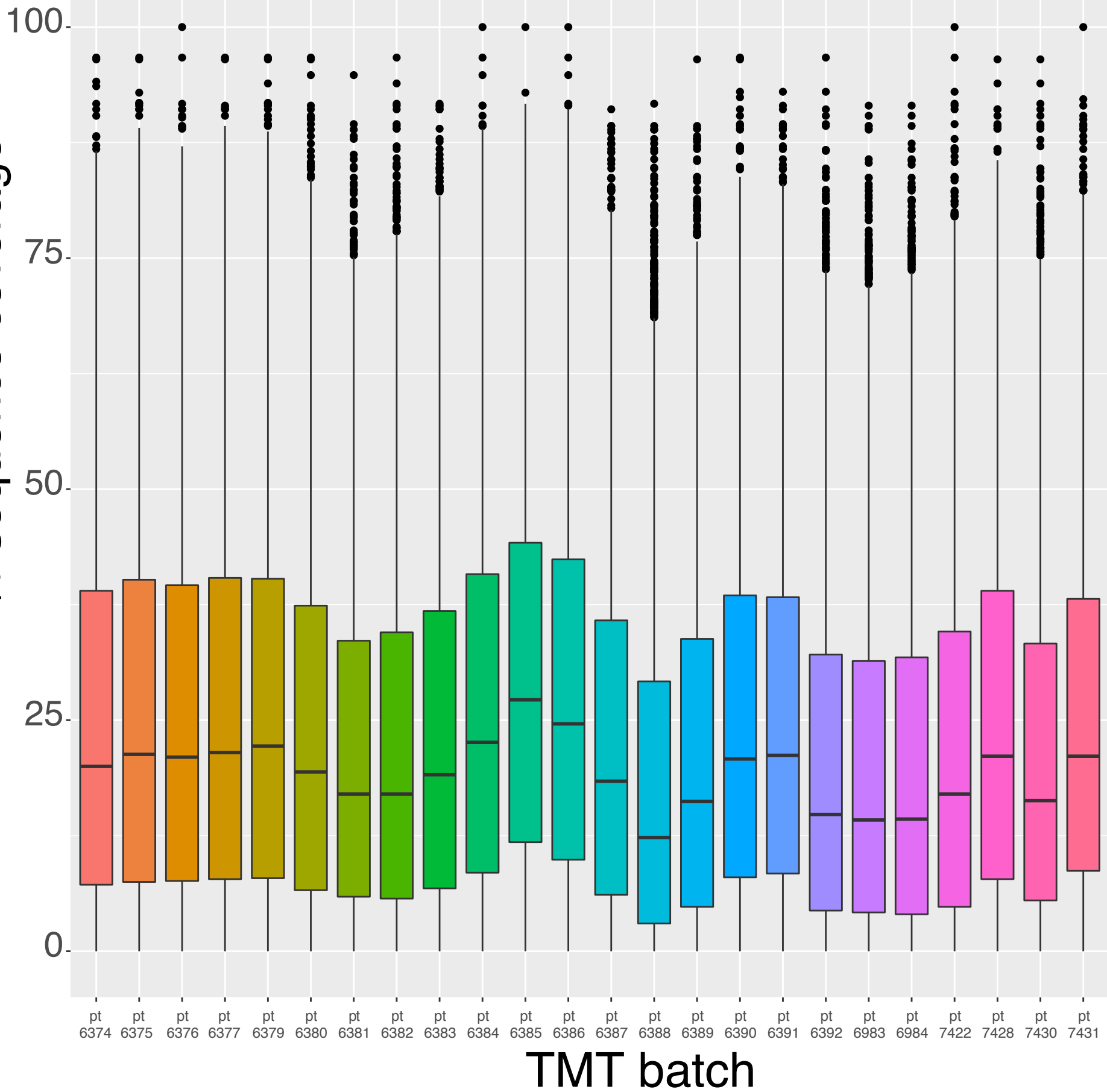

Supplement: Sequence coverage acros batches [file 144550_2_supp_346179_psxjcq.pdf]

batch

|        |        |        |        |        |
|--------|--------|--------|--------|--------|
| pt6374 | pt6380 | pt6384 | pt6388 | pt6984 |
| pt6375 | pt6381 | pt6385 | pt6389 | pt7422 |
| pt6377 | pt6382 | pt6386 | pt6391 | pt7428 |
| pt6379 | pt6383 | pt6387 | pt6983 | pt7431 |

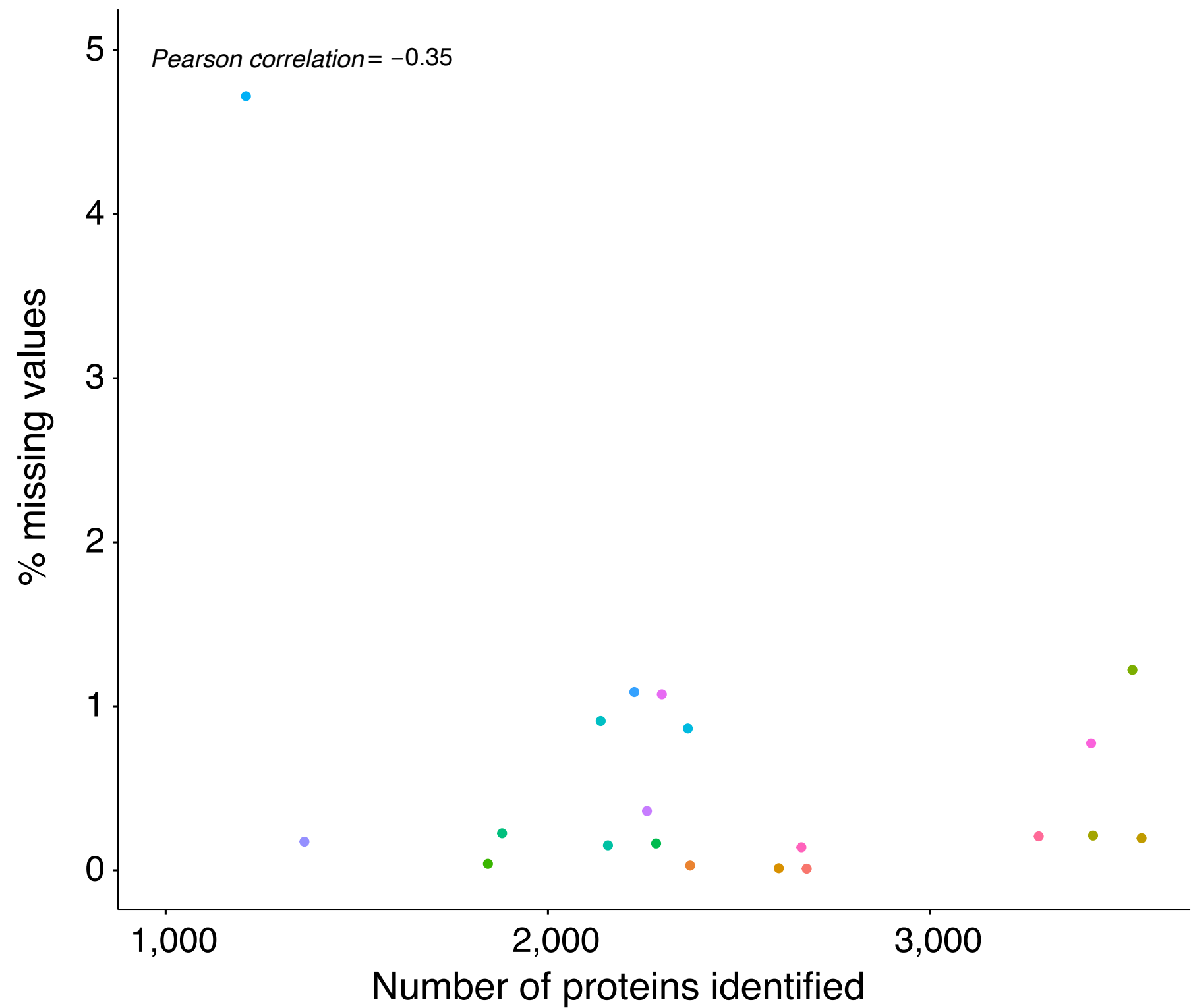

Supplement: QC vs Mising values [file 144550_2_supp_346187_psxj1r.pdf]
