# Supplementary material for: Multibatch TMT Reveals False Positives, Batch Effects and Missing Values
Source: Mol Cell Proteomics. 2019 Jul 22;18(10):1967–80. doi: 10.1074/mcp.RA119.001472 (PMC6773557; doi:10.1074/mcp.RA119.001472)
Supplement: Number of proteins identified [file 144550_2_supp_346178_psxjmq.pdf]

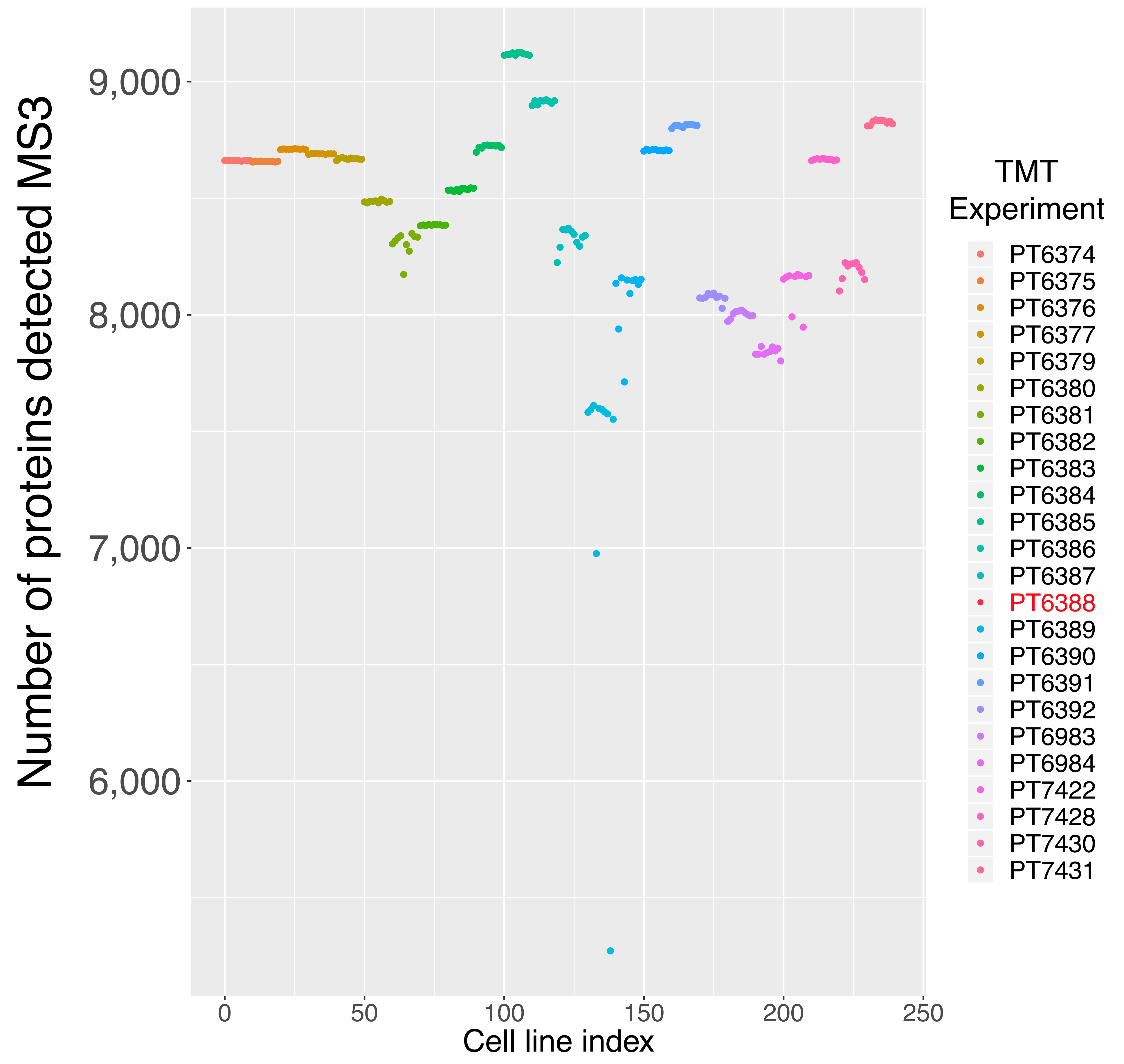
Number of proteins detected MS3
9,000
8,000
7,000
6,000
0
50
100
150
200
250
Cell line index
TMT Experiment
PT6374
PT6375
PT6376
PT6377
PT6379
PT6380
PT6381
PT6382
PT6383
PT6384
PT6385
PT6386
PT6387
PT6388
PT6389
PT6390
PT6391
PT6392
PT6983
PT6984
PT7422
PT7428
PT7430
PT7431
