# Supplementary material for: Multibatch TMT Reveals False Positives, Batch Effects and Missing Values
Source: Mol Cell Proteomics. 2019 Jul 22;18(10):1967–80. doi: 10.1074/mcp.RA119.001472 (PMC6773557; doi:10.1074/mcp.RA119.001472)
Supplement: QC vs Proteins MS2 [file 144550_2_supp_346188_psxjnr.pdf]

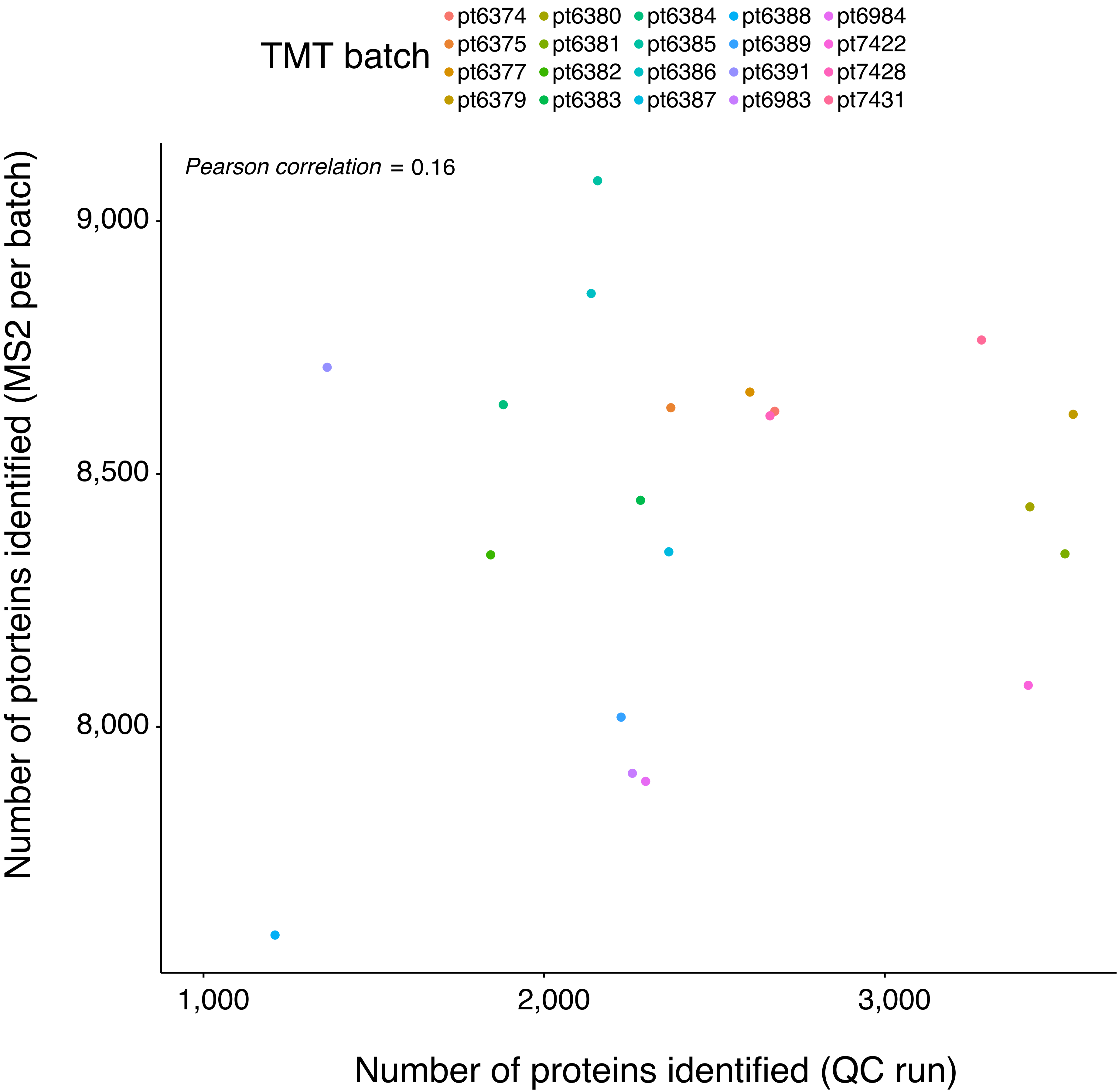
TMT batch
pt6374
pt6375
pt6377
pt6379
pt6380
pt6381
pt6382
pt6383
pt6384
pt6385
pt6386
pt6387
pt6388
pt6389
pt6391
pt6983
pt6984
pt7422
pt7428
pt7431
Pearson correlation = 0.16
9,000
8,500
8,000
Number of ptorteins identified (MS2 per batch)
1,000
2,000
3,000
Number of proteins identified (QC run)
